# An Abrupt Centennial-Scale Drought Event and Mid-Holocene Climate Change Patterns in Monsoon Marginal Zones of East Asia

**DOI:** 10.1371/journal.pone.0090241

**Published:** 2014-03-05

**Authors:** Yu Li, Nai'ang Wang, Chengqi Zhang

**Affiliations:** College of Earth and Environmental Sciences, Center for Hydrologic Cycle and Water Resources in Arid Region, Lanzhou University, Lanzhou, China; Agharkar Research Institute, India

## Abstract

The mid-latitudes of East Asia are characterized by the interaction between the Asian summer monsoon and the westerly winds. Understanding long-term climate change in the marginal regions of the Asian monsoon is critical for understanding the millennial-scale interactions between the Asian monsoon and the westerly winds. Abrupt climate events are always associated with changes in large-scale circulation patterns; therefore, investigations into abrupt climate changes provide clues for responses of circulation patterns to extreme climate events. In this paper, we examined the time scale and mid-Holocene climatic background of an abrupt dry mid-Holocene event in the Shiyang River drainage basin in the northwest margin of the Asian monsoon. Mid-Holocene lacustrine records were collected from the middle reaches and the terminal lake of the basin. Using radiocarbon and OSL ages, a centennial-scale drought event, which is characterized by a sand layer in lacustrine sediments both from the middle and lower reaches of the basin, was absolutely dated between 8.0–7.0 cal kyr BP. Grain size data suggest an abrupt decline in lake level and a dry environment in the middle reaches of the basin during the dry interval. Previous studies have shown mid-Holocene drought events in other places of monsoon marginal zones; however, their chronologies are not strong enough to study the mechanism. According to the absolutely dated records, we proposed a new hypothesis that the mid-Holocene dry interval can be related to the weakening Asian summer monsoon and the relatively arid environment in arid Central Asia. Furthermore, abrupt dry climatic events are directly linked to the basin-wide effective moisture change in semi-arid and arid regions. Effective moisture is affected by basin-wide precipitation, evapotranspiration, lake surface evaporation and other geographical settings. As a result, the time scales of the dry interval could vary according to locations due to different geographical features.

## Introduction

The Earth's climate zones are classified according to their average temperature and rainfall accumulation, and, in general, form latitudinal, east-west oriented bands on the Earth's surface [1], [2]. The mid-latitude climate is affected by two different air-masses, which are cold air masses from the poles and warm air masses from the tropics. In East and Central Asia, the mid-latitude climate is characterized by the interaction between the Asian summer monsoon from the Tropical Ocean and the westerly winds from the mid-to-high latitudes [3], [4]. Furthermore, the interaction between the monsoon and the westerly winds is influenced by the Qinghai-Tibet Plateau, which is a natural obstacle for blocking the interaction between the air masses from different latitudes [5], [6]. Various climatic zones are distributed in the mid-latitude regions of East and Central Asia [5].

During the Holocene epoch, there are many uncertainties relating to the Holocene climate change in the mid-latitude regions of East and Central Asia, and the climate change processes may differ in different parts of the region [7]–[11]. Li (1990) suggested the climate in northwest China could be divided into monsoon and westerly wind patterns in terms of the millennium-scale climate change [12]. Herzschuh (2006) synthesized 75 records for the Asian continent and found that the Holocene climate patterns differed between Central Asia and some parts of the Asian monsoon domain [13]. Chen et al. (2008) analyzed the absolutely dated Holocene records for the Asian monsoon domain and arid Central Asia and found that Holocene millennial-scale climate patterns are different for the two regions [14]. Chen et al. (2008) also concluded the millennium-scale difference is mainly due to different evolution histories of the Asian monsoon and westerly winds [14]. Since the Asian summer monsoon reached their highest level during the early Holocene (ca. 9000 cal yr BP), while the westerly winds were relatively dry during the early Holocene on the millennial-scale [13], [14]. According to previous studies, the millennial-scale interaction between the Asian summer monsoon and the westerly winds could be a reason for asynchronous Holocene climate changes in East and Central Asia.

**Figure 1:**
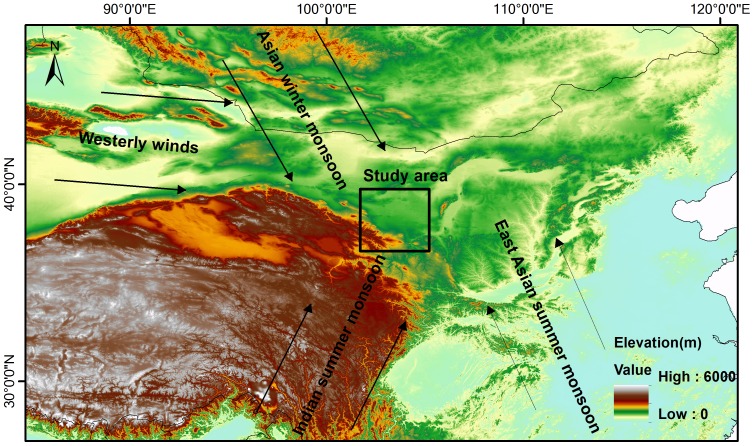
Map showing the study area and the arrows indicate the climate systems affecting China, including the East Asian summer monsoon, the Indian summer monsoon, the Asian winter monsoon, and the westerly winds.

**Figure 2:**
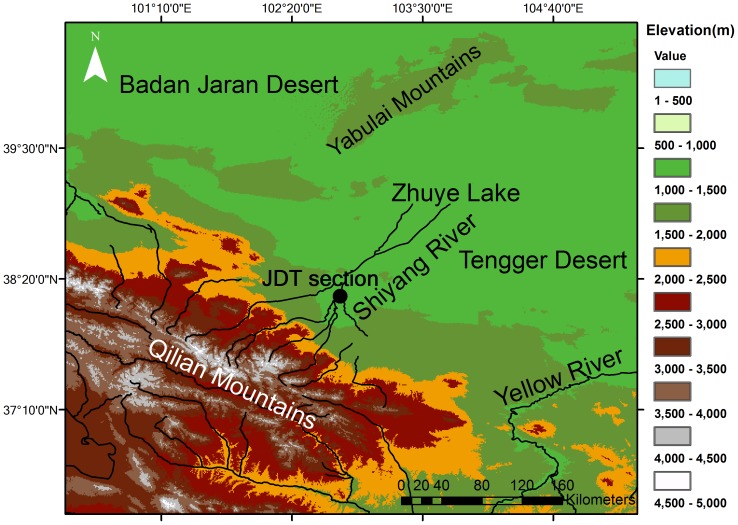
Map showing the topography around Zhuye Lake and the Shiyang River drainage basin. The black circles indicate the JDT section.

Monsoon marginal zones in East Asia are affected both by the Asian monsoon and the westerly winds [5], [6]. Abrupt climatic changes during the mid-Holocene records have been reported in different areas of monsoon marginal zones in East and Central Asia: Mongolia [15], [16], the Alashan Plateau [17], central Inner Mongolia [18], eastern Inner Mongolia [19], the Loess Plateau [20]. The mid-Holocene dry interval was also reported in southern China [21]. The time scales and mechanisms of abrupt climate change events in monsoon marginal zones are crucial for understanding the millennial-scale interaction between the Asian monsoon and the westerly winds. Two kinds of hypotheses have been proposed regarding the mid-Holocene abrupt climate events: one is relating the abrupt dry mid-Holocene interval to the increased evaporation [18], [22]; the other links the interval to the weakened Asian summer monsoon [17], [19]. However, restricted by the ages of the records, the mechanisms about the abrupt mid-Holocene intervals are still in dispute. Absolutely dated mid-Holocene climate records are needed for investigations into abrupt mid-Holocene climatic changes. The Shiyang River drainage basin is located in the northwest margin of the Asian monsoon (Figure 1, Figure 2). Previous studies have reported long-term Holocene environmental changes by the palynology and geochemical proxies [17], [23]–[27]. In relatively weak chronological frames, it is difficult to study the mechanisms of the abrupt mid-Holocene climate events [25]–[27]. Chen et al. (2003, 2006) have linked the mid-Holocene abrupt changes to the weakening Asian summer monsoon; however, this hypothesis cannot be supported by the gradual change of the mid-Holocene Asian monsoon [17], [23], [28], [29]. The time scales and mechanisms of the abrupt climatic events during the mid-Holocene are still unclear in this area, which is a key area for studying the millennial-scale interaction between the Asian summer monsoon and the westerly winds. In this study, we used chronological and grain size data from QTH01 and QTH02 sections in the terminal lake. Radiocarbon and OSL dates of the sand layers were compared to study the chronologies, while grain size data provide more details about the environments during the abrupt climate change. In addition, mid-Holocene lacustrine sediments (JDT section) from the middle reaches of the basin were also studied for a basin-wide comparison. According to chronologies of the mid-Holocene sediments from different parts of the basin, we proposed a new hypothesis regarding the mid-Holocene climate change patterns in monsoon marginal zones.

## Geographical Setting

The Shiyang River basin is located at the east part of the Hexi Corridor of Gansu Province in northwest China. It is on the northern slope of Qilian Mountain at longitude of 101°41′–104°16′E and latitude of 36°29′–39°27′N (Figure 2). The river originates from the southern part of the mountain and ends at the terminal lake, Zhuye Lake. The whole river basin has an area of 41,600 km^2^. The river basin spans over three climatic zones from south to north. The south cold semi-arid to semi-humid zone at the highland of Qilian Mountain (altitude 2000–5000 m) has an annual precipitation of 300–600 mm and pan evaporation of 700–1200 mm. The vegetation zones can be divided into the alpine cushion zone, alpine meadow zone, alpine shrub zone, forest zone, and piedmont meadow steppe zone. The middle cool arid zone at the flatland of Hexi Corridor (altitude 1500–2000 m) has an annual precipitation of 150–300 mm and pan evaporation of 1200–2000 mm. The vegetation zone is a desert steppe zone. The north temperate arid zone (altitude 1300–1500 m) has an annual precipitation of less than 150 mm and pan evaporation of more than 2000 mm. Desert vegetation is widely distributed in this area [30], [31]. Around the terminal lake and near the edge of the Tengger desert, the annual rainfall is about 50 mm and pan evaporation is 2000–2600 mm. Based on the geographical divisions in China, the Shiyang River drainage basin is in the transition zone of monsoonal and arid regions, and the modern climate of the region is affected by the Asian monsoon and the westerly winds [5]. According to modern climate research, the westerly wind comprises a prevailing westerly wind and a westerly jet, both of which make a large contribution to water vapor transportation in the basin. In the alpine areas of the Shiyang River basin, the summer precipitation is also contributed by the Asian summer monsoon [4]. The terminal lake, Zhuye Lake, has been dried up since the 1950s, a result of the diversion of water from the Shiyang River for irrigation and other purposes. Based on the geomorphologic studies, the lake level is relatively high during the historical period, the Marine Isotopic Stage 3 (MIS3) and the early Holocene periods [32]–[34]. At present, the dry lakes that exist in several depressions on the Tengger desert margins only fill with water in years with sufficient precipitation.

## Materials and Methods

**Figure 3:**
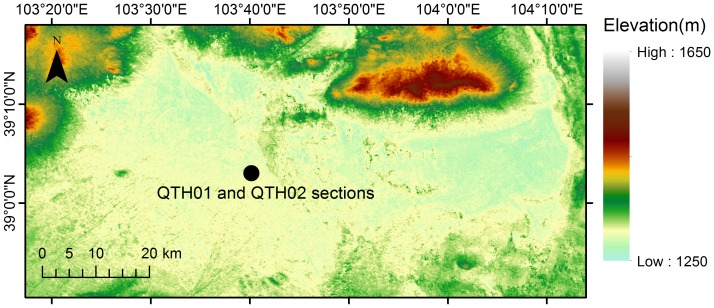
Map showing latitudes, longitudes and elevations of the Zhuye Lake basin. The black circle indicates the location of the QTH01 and QTH02 sections. (The elevation data are based on the ASTER-GDEM dataset, with 30 m resolution).

**Figure 4:**
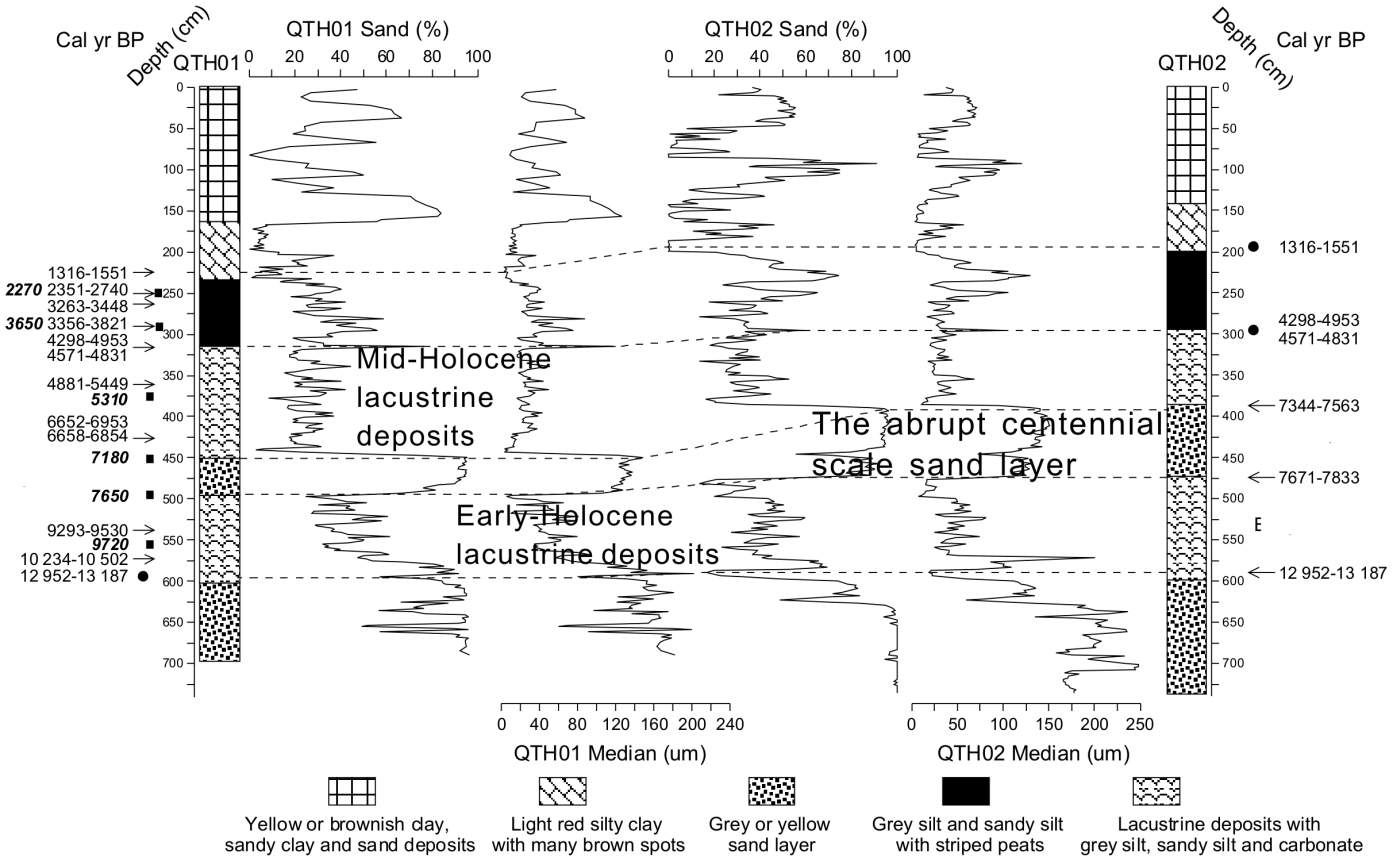
Lithology and ages in the QTH01 and QTH02 sections. Subsurface correlation of the QTH01 and QTH02 sections, based on grain size data (% sand and Median (µm)) plotted against depth. Of the 14 radiocarbon dates (Table 1), 11 are from QTH01, the remainder from QTH02. All the OSL dates are from QTH01 (Table 2). The arrowheads show the radiocarbon dates, and the black rectangles show the OSL dates. The OSL dates are highlighted using italics. The broken lines connect the extrapolated ages between two sections and the extrapolated ages are marked with black circles. During the mid-Holocene three lithologic phases are showed beside the lithologic columns, including the mid-Holocene lacustrine deposits, the abrupt centennial-scale dry interval and the early-Holocene lacustrine deposits.

In the terminal lake, the QTH01 and QTH02 sections were excavated in the central part of Zhuye Lake. No specific permissions were required for these excavation works. The field studies did not involve endangered or protected species and the specific location can be seen below. The two sections are at an elevation of 1309 m above sea level. The geographic coordinates are 39°03′00′′N, 103°40′08′′E (Figure 3). Stratigraphically (Figure 4), QTH01 at 0–165 cm are aeolian sediments, composed of yellow and brownish clay, sandy clay and sand deposits, influenced by agricultural activities on the top of the section; 165–230 cm are alluvial sediments, composed of light red silty clay with many brown spots; 230–315 cm are lacustrine deposits formed in shallow lake composed of grey silt and sandy silt, and striped peats with plant fragments and mollusk shells are embedded; 315–450 cm are typical lacustrine deposits with grey silt, sandy silt and carbonate; 450–495 cm is a grey sand layer with some cracked mollusc shells; 495–603 cm is a lacustrine deposit with grey, carbonate enriched silt; 603–692 cm is grey or yellow sand and well sorted. QTH02 shows a similar stratigraphy (Figure 4). QTH01 was sampled at 2 cm intervals at the lake sediment layers and at 5 cm intervals otherwise, resulting in 292 samples for analyses of grain size. QTH02 was sampled at 2 cm intervals, yielding 368 samples for analysis of grain size. The JDT section (38°10′46″N, 102°45′53″E) is located at an elevation of 1460 m asl (Figure 2). The JDT section is 580 cm thick, and a total of 58 samples are obtained and for analyzing grain size. Stratigraphically (Figure 3), JDT at 0–120 cm are aeolian deposits, composed of yellow or brown silty clay; 120–230 cm are a kind of lacustrine deposits, composed of grey carbonate enriched silty clay or silt with plant fragments; 230–300 cm is a light yellow sand layer with many brown spots; 300–330 cm is silt peat with plant fragments; 330–400 cm is grey or brown silty clay or silt with roots and carbonate; 400–580 cm is yellow or grey gravel sand with roots.

**Figure 5:**
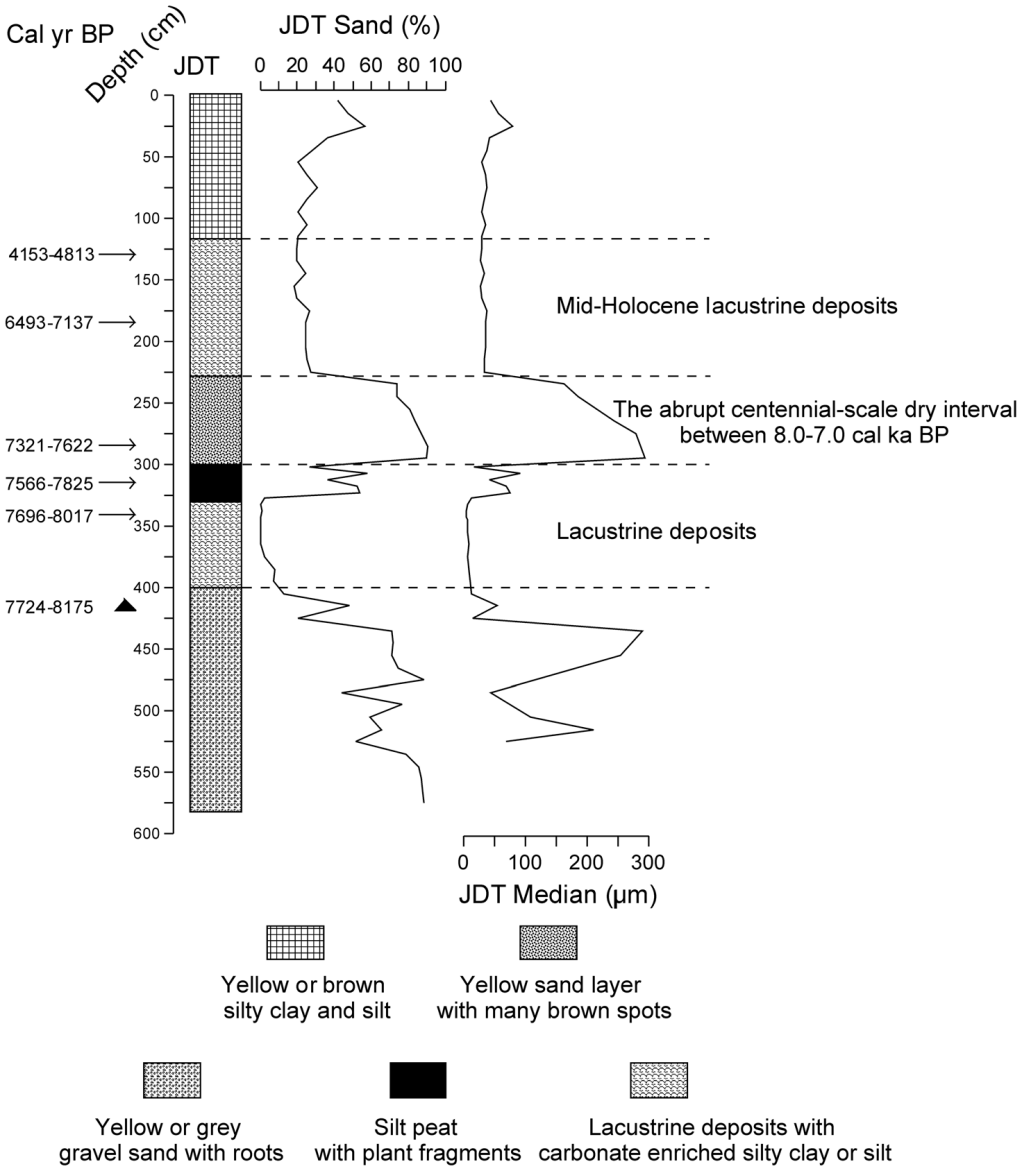
Lithology and ages in the JDT section. Of the 6 radiocarbon dates (Table 1), 5 are dated by bulk samples, and the remainder is dated by terrestrial branches. The arrowheads show the bulk samples' dates, and the black triangle shows the date of the terrestrial branches. During the mid-Holocene, three lithologic phases are showed beside the lithologic column, including the mid-Holocene lacustrine deposits, the abrupt centennial-scale dry interval and the lacustrine deposits below the dry interval.

In the QTH01 and QTH02 sections, fourteen radiocarbon dates are obtained. Six of them are dated in Peking University Dating Laboratory, others are dated in Lanzhou University Dating Laboratory. Of the 14 radiocarbon dates (Figure 4, Table 1), 11 are from QTH01, the remainder from QTH02. Dates were calibrated using CALIB 6.11 and are reported in calibrated years. For correcting the hard water effects, six optically stimulated luminescence (OSL) samples are dated to provide chronological control at QTH01 section (Figure 4, Table 2) [27]. Quartz (38–63 µm) OSL measurements were performed in the Luminescence Dating Laboratory of the Qinghai Institute of Salt Lakes, Chinese Academic Sciences, using an automated Risø TL/OSL-20 reader. In arid/semiarid regions of China, previous studies showed that radiocarbon dating of lake sediments is likely affected by the hard water effects [35]. For estimating the reliability of ^14^C dating, we compare the calibrated radiocarbon ages with OSL ages adjusted on AD 1950, at QTH01 section (Figure 4, Table 1, Table 2). The OSL and ^14^C ages are in good agreement, suggesting that the established chronology is robust. Beside the comparison between the OSL dates and the radiocarbon dates, as shown in Table 1, the age difference of the radiocarbon date for organic matter and the date for shells at the same depth (3.15 m) is only ∼30 years. Based on these results, we concluded the hard water effects were slight in the two sections. Most stratigraphic units are correlated between the two sections. After analyzing lithology and grain size in the two sections, the radiocarbon and OSL dates can be compared from the two sections (Figure 4). The chronology of the JDT section is based on six radiocarbon dates of organic matter and terrestrial branches analyzed in Lanzhou University Dating Laboratory (Table 1, Figure 5). In the JDT section, at the depth of 4.20 m, the dating material is terrestrial branches, which are less influenced by the hard water effects. Based on a comparison between ages from terrestrial branches and organic matter, the hard water effect is also slight in the middle reaches of the Shiyang River drainage basin. Grain size distribution was determined with a Malvern Mastersizer 2000 particle analyzer that automatically yields the percentages of clay-, silt- and sand-size fractions, as well as median, mean and mode sample diameters. 0.2–0.4 g of sediment was pretreated by heating in 10 ml of 10% H_2_O_2_ to remove organics, heated in 10 ml 10% HCl to remove carbonate that otherwise would bond different mineral fractions, then shaken in Na-hexametaphosphate to disaggregate the sediment for 1 h prior to analysis. The median grain size and the grain size frequency curves are all based on the sample diameters. Sand percentages are based on sediment fractions that are higher than 63 µm.

**Table 1:** AMS and conventional radiocarbon ages in the QTH01, QTH02 and JDT sections.

Laboratory number	Depth in section (m)	Dating material	^14^C age (yr BP)	Calibrated ^14^c age (2σ)(cal yr BP)
	QTH01			
LUG96-44	2.25	Organic matter	1550±60	1316–1551
LUG96-45	2.5	Organic matter	2470±90	2351–2740
BA05223	2.62	shells	3140±40	3263–3448
LUG96-46	2.9	Organic matter	3300±90	3356–3821
LUG96-47	3.15	Organic matter	4130±110	4298–4953
BA05224	3.15	shells	4160±40	4571–4831
LUG96-48	3.6	Organic matter	4530±80	4881–5449
LUG96-49	4.25	Inorganic matter	5960±65	6652–6953
BA05225	4.25	shells	5920±40	6658–6854
LUG02-25	5.37	Organic matter	8412±62	9293–9530
LUG02-23	5.72	Organic matter	9183±60	10234–10502
	QTH02			
BA05222	3.88	shells	6550±40	7344–7563
BA05221	4.75	shells	6910±40	7671–7833
BA05218	5.91	shells	11175±50	12952–13187
	JDT			
LUG96-53	1.33	Organic matter	3980±96	4153–4813
LUG96-51	1.87	Organic matter	5930±100	6493–7137
LUG96-54	2.84	Organic matter	6600±90	7321–7622
LUG96-50	3.15	Organic matter	6820±70	7566–7825
LUG96-55	3.4	Organic matter	7060±85	7696–8017
LUG96-52	4.2	Terrestrial branches	7130±110	7724–8175

**Table 2:** OSL dates for six samples in the QTH01 section [27]. All the OSL dates were adjusted to AD 1950 to compare with calibrated ^14^C dates.

Depth (cm)	U (ppm)	Th (ppm)	K (%)	Water content (%)	Dose rate (Gy/kyr)	De (Gy)	Age (kyr)
250	11.19±0.35	2.65±0.16	0.58±0.03	50±5	2.02±0.16	4.70±0.10	2.27±0.19
290	6.27±0.27	4.34±0.20	0.97±0.04	45±5	1.76±0.13	6.50±0.20	3.65±0.30
375	4.08±0.21	1.70±0.14	0.32±0.02	58±5	0.76±0.07	4.10±0.30	5.31±0.60
455	4.47±0.23	2.93±0.18	1.30±0.05	44±5	1.60±0.12	11.80±0.50	7.18±0.63
495	8.10±0.28	2.82±0.17	0.97±0.03	57±5	1.49±0.12	11.50±0.30	7.65±0.67
560	10.65±0.35	5.48±0.25	1.17±0.04	51±5	2.21±0.18	21.60±0.50	9.72±0.81

## Chronological and Grain Size Data for the Abrupt Dry Mid-Holocene Interval

### 1 Ages of the mid-Holocene sand layers in the QTH01, QTH02 and JDT sections

**Figure 6:**
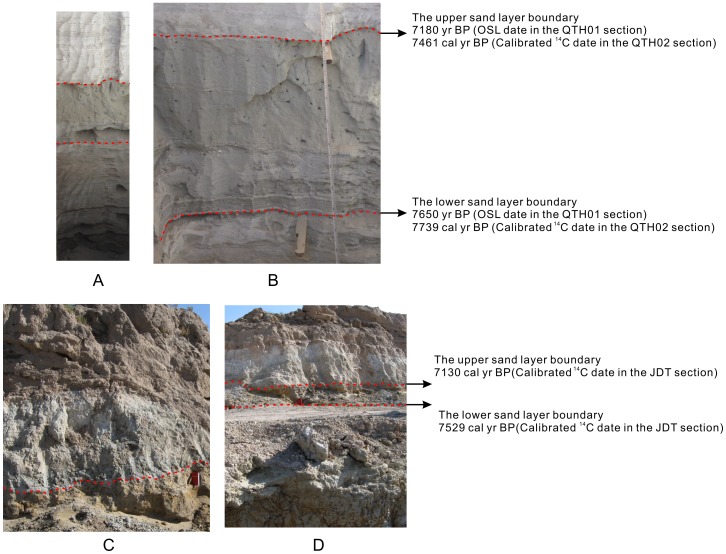
Pictures showing the mid-Holocene sand layers and the dates in the QTH02 and JDT sections. The red broken lines indicate the boundaries of the mid-Holocene sand layers in the QTH02 and JDT sections. A and B show the sand layer in the QTH02 section. C and D show the sand layer in the JDT section.

In the QTH01 section, the mid-Holocene sand layer is dated by OSL dating method. The upper boundary is around 7180 years BP, and the lower boundary is around 7650 years BP (Table 2). In the QTH02 section, the mid-Holocene sand layer is dated by radiocarbon dating method. The upper boundary is around 7461 cal yr BP, and the lower boundary is around 7739 cal yr BP (Figure 6, Table 2). In the JDT section, linear interpolated by the mid-Holocene radiocarbon dates, the upper boundary of the sand layer is 7130 cal yr BP, and the lower boundary is around 7529 cal yr BP (Figure 6, Table 1). Totally, based on the radiocarbon and OSL dates of the three sections in the Shiyang River drainage basin, the abrupt dry mid-Holocene interval appears to be a centennial-scale dry interval between 8.0–7.0 cal kyr BP.

### 2 Grain size data of the mid-Holocene sand layers

**Figure 7:**
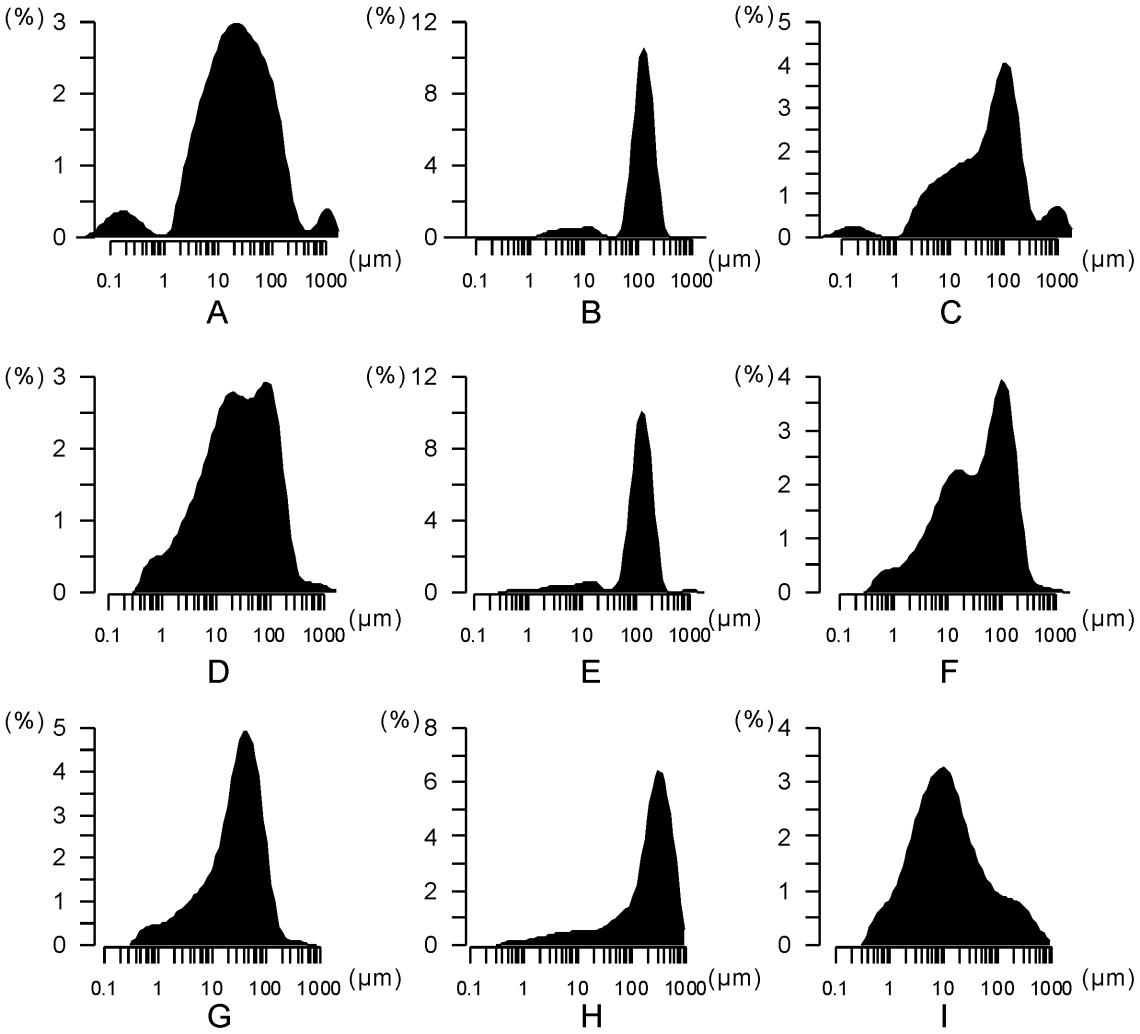
Average grain size frequency distribution curves in the QTH01 and QTH02 and JDT sections. A, B and C represent the three mid-Holocene phases, including the mid-Holocene lacustrine deposits, the abrupt centennial-scale dry interval and the early-Holocene lacustrine deposits in the QTH01 section. D, E and F represent the three mid-Holocene phases, including the mid-Holocene lacustrine deposits, the abrupt centennial-scale dry interval and the early-Holocene lacustrine deposits in the QTH02 section. G, H and I represent the three mid-Holocene phases, including the mid-Holocene lacustrine deposits, the abrupt centennial-scale dry interval and the lacustrine deposits below the dry interval in the JDT section.

It is described as the sediment sorting principle that the grain size of lake sediments becomes finer and finer from the shore to the center, and sediment belts of different grain size fractions levels can be distinguished, as a result, the grain size of lake sediments is in close relation to water level and the energy of the inflows [26], [36]. According to this theory, grain size change in the QTH01 and QTH02 sections of Zhuye Lake can be related to the lake level change. The general mid-Holocene grain size change can be shown by sand percentages (>63 µm) and median grain size (Figure 4). Mid-Holocene sediments can be divided into three phases according to the grain size data from QTH01 and QTH02 sections (Figure 4, Figure 7): the mid-Holocene lacustrine deposits, the abrupt centennial-scale sand layer and the early-Holocene lacustrine deposits. In the QTH01 section, the mid-Holocene lacustrine deposits exist at the depth of 315–450 cm (∼7.2–∼4.7cal kyr BP). During this phase, the average sand percentage is 25.73% (6.18–68.43%), and the average median grain size is 37.24 µm (0.38–107.89 µm) (Figure 4). The average grain size frequency distribution curve can be divided into 3 parts: 0.03–0.89 µm, 0.89–447.74 µm and 447.74–1782.50 µm. There are also three peaks (0.18 µm, 22.44 µm and 1124.68 µm, relatively) corresponding to the three parts (Figure 7). The abrupt centennial-scale sand layer appears between at the depth of 450–495 cm (∼7.6–∼7.2cal kyr BP). During this phase, the average sand percentage is 88.24% (65.95–95.48%), and the average median grain size is 128.01 µm (105.30–146.74 µm). The average grain size frequency distribution curve can be divided into 2 parts: 0.05–35.57 µm and 35.57–447.74 µm. Two peaks for the two parts are 12.62 µm and 126.19 µm, respectively. The early-Holocene lacustrine deposits appear at the depth of 495–600 cm (∼13.0–∼7.6 cal kyr BP). During this phase, the average sand percentage content is 50.51% (12.26–91.50%), and the average median grain size is 77.66 µm (2.51–230.94 µm). The average grain size frequency distribution curve can be divided into 4 parts: 0.04–0.89 µm, 0.89–28.25 µm, 28.25–447.74 µm and 447.74–1782.50 µm.

In the QTH02 section, the mid-Holocene lacustrine deposits appear at the depth of 297-385 cm (∼7.4–∼4.7 cal kyr BP). During this phase, the average sand percentage is 31.24% (13.52–52.75%), and the average median grain size is 30.73 µm (11.28–68.69 µm) (Figure 4). The average grain size frequency distribution curve can be divided into 3 parts: 0.36–1.00 µm, 1.00–399.05 µm and 399.05–1782.50 µm (Figure 7). The abrupt centennial-scale sand layer appears at the depth of 385–475 cm (∼7.7–∼7.4 cal kyr BP). The average sand percentage is 87.68% (47.18–96.22%), and the average median grain size is 127.86 µm (55.41–151.23 µm) during this phase. The average grain size frequency distribution curve can be divided into 3 parts: 0.36–31.70 µm, 31.70–400.00 µm and 400.00–1782.50 µm, and there are three peaks (14.16 µm, 126.19 µm and 1002.37 µm relatively) corresponding to the three parts. The early-Holocene lacustrine deposits appear between at the depth of 475–599 cm (∼13.0–∼7.7 cal kyr BP). During this phase, the average sand percentage is 41.45% (13.55–96.16%), and the average median grain size is 49.74 µm (7.4–200.81 µm). The average grain size frequency distribution curve can be divided into 4 parts, 0.36–1.12 µm, 1.12–31.70 µm, 31.70–316.98 µm and 316.98–1415.89 µm.

In the middle reaches of the basin, the JDT section is located on a terrace of the Hongshui River, which is a tributary of the Shiyang River. The sedimentary facies and grain size of the JDT section are mostly controlled by the environmental conditions in the middle reaches of the basin. Located beside the Tengger Desert, lacustrine sediments and peat are formed during the humid period; on the contrary, sediments are dominated by sand and aeolian sediments during the arid period. Based on the grain size data (Figure 5), the mid-Holocene sediments can be divided into three phases: the mid-Holocene lacustrine deposits, the abrupt centennial-scale sand layer and the lacustrine deposits below the sand layer (Figure 5, Figure 7). In the JDT section, the mid-Holocene lacustrine deposits appear between at the depth of 120–230 cm (∼7.1–∼4.4 cal kyr BP). During this phase, the average sand percentage is 23.23% (18.67–27.16%), and the average median grain size is 33.14 µm (27.26–37.90 µm) (Figure 5). The average grain size frequency distribution curve can be divided into 3 parts, 0.36–1.12 µm, 1.12–200.00 µm and 200.00–563.68 µm (Figure 7). The abrupt centennial-scale sand layer appears at the depth of 230–300 cm (∼7.5–∼7.1 cal kyr BP). During this phase, the average sand percentage is 83.00% (73.99–90.54%), and the average median grain size is 264.88 µm (164.09–372.61 µm). The average grain size frequency distribution curve can be divided into 2 parts: 0.40–20.00 µm and 20.00–893.37 µm. The lacustrine deposits below the sand layer appear at the depth of 300–400 cm (∼7.9–∼7.5 cal kyr BP). During this phase, the average sand percentage is 16.41% (0.13–58.17%), and the average median grain size is 264.88 µm (164.09–372.61 µm). The average grain size frequency distribution curve can be divided into 3 parts: 0.36–1.26 µm, 1.26–89.34 µm and 89.34–893.37 µm.

In the QTH01, QTH02 and JDT sections, grain size data indicate that sediments from the mid-Holocene sand layers are much coarser than those of the mid-Holocene lacustrine deposits from different parts of the basin. In the terminal lake, mid-Holocene environmental changes implied by QTH01 and QTH02 sections are closely related to lake level changes, while the abrupt sand layer can show an abrupt decline in lake level. In the JDT section, the coarser deposits from the mid-Holocene sand layer could indicate the arid environment and the expansion of the desert in the middle reaches of the basin. The average grain size frequency distribution curves of the sand layers from the QTH01, QTH02 and JDT sections are similar to each other (Figure 7), showing the deposits are well sorted with a symmetrical distribution and mainly distributed between 100 and 300 µm. This kind of grain size frequency distribution is similar to that of the desert surface samples in the surrounding regions, northern China [37]. This could indicate that the deposits of the mid-Holocene sand layers are mainly from the surrounding deserts. In the three sections, grain size of the mid-Holocene lacustrine deposits can be generally divided into 3 or more than 3 parts and poorly sorted. The relatively fine components, below 40 µm, generally account for a large fraction, which can be the suspended load [37], [38]. At the same time, lacustrine deposits from arid regions can be transported by many dynamic processes, such as wind transportation, saltation and suspension transportations in water [37]. The poorly sorted grain size data and the characteristics of grain size frequency distribution show multiple transport powers in the region.

## Discussion

Based on the radiocarbon and OSL dates of the mid-Holocene sand layers from QTH01, QTH02 and JDT sections in the Shiyang River drainage basin, the abrupt centennial-scale dry interval is between 8.0 and 7.0 cal kyr BP, and the abrupt sand layer is embedded between the early and middle Holocene lacustrine sediments. The sedimentary sequences show that the relatively humid early and middle Holocene was interrupted by an abrupt dry event in the basin. In the west of Zhuye Lake, Chen et al. (2003) reported a dry middle Holocene interval (∼7.0–∼5.0 cal kyr BP) [17]. In the middle reaches of the Shiyang River, Zhang et al. (2000) also found a mid-Holocene sand layer (∼6.3–∼5.7 cal kyr BP) [39]. However, previous studies failed to establish an absolutely dated mid-Holocene climate change record due to a relatively loose age control. Based on a comparison between OSL and radiocarbon ages in the terminal lake and the mid-Holocene record in the middle reaches of the basin, this work provide an accurate chronological framework for an abrupt dry mid-Holocene interval that happened between 8.0 and 7.0 cal kyr BP, while the grain size data indicate a low lake level in the terminal lake and an arid environment in the middle reaches during the abrupt dry interval. The 8.2 kyr BP event (the major cooling episode 8200 years before present) has been widely regarded as the most remarkable cooling episode during the early Holocene [40]. Although the radiocarbon and the OSL dating methods have some uncertainties on the centennial-scale, the abrupt dry event recovered from the Shiyang River Basin was dated by different dating methods and found at different sites of the drainage basin. All the ages at the boundaries of the sand layers indicate that the dry interval is not beyond 8.0 cal kyr BP. Furthermore, early Holocene records in surrounding areas did not show an obvious cool/dry signal of the 8.2 kyr BP event [24], [27]. As a result, the abrupt dry interval between 8.0–7.0 cal kyr BP cannot be linked to the 8.2 kyr BP event directly. This drainage basin is located in the northwest margin of the Asian summer monsoon, where the modern climate is affected both by the Asian summer monsoon and the westerly winds. A comparative study between this record and mid-Holocene climatic records from the Asian monsoon and westerly winds domains is critical for understanding the mechanism of the abrupt dry interval.

### 1 Mid-Holocene Asian summer monsoon evolution

**Figure 8:**
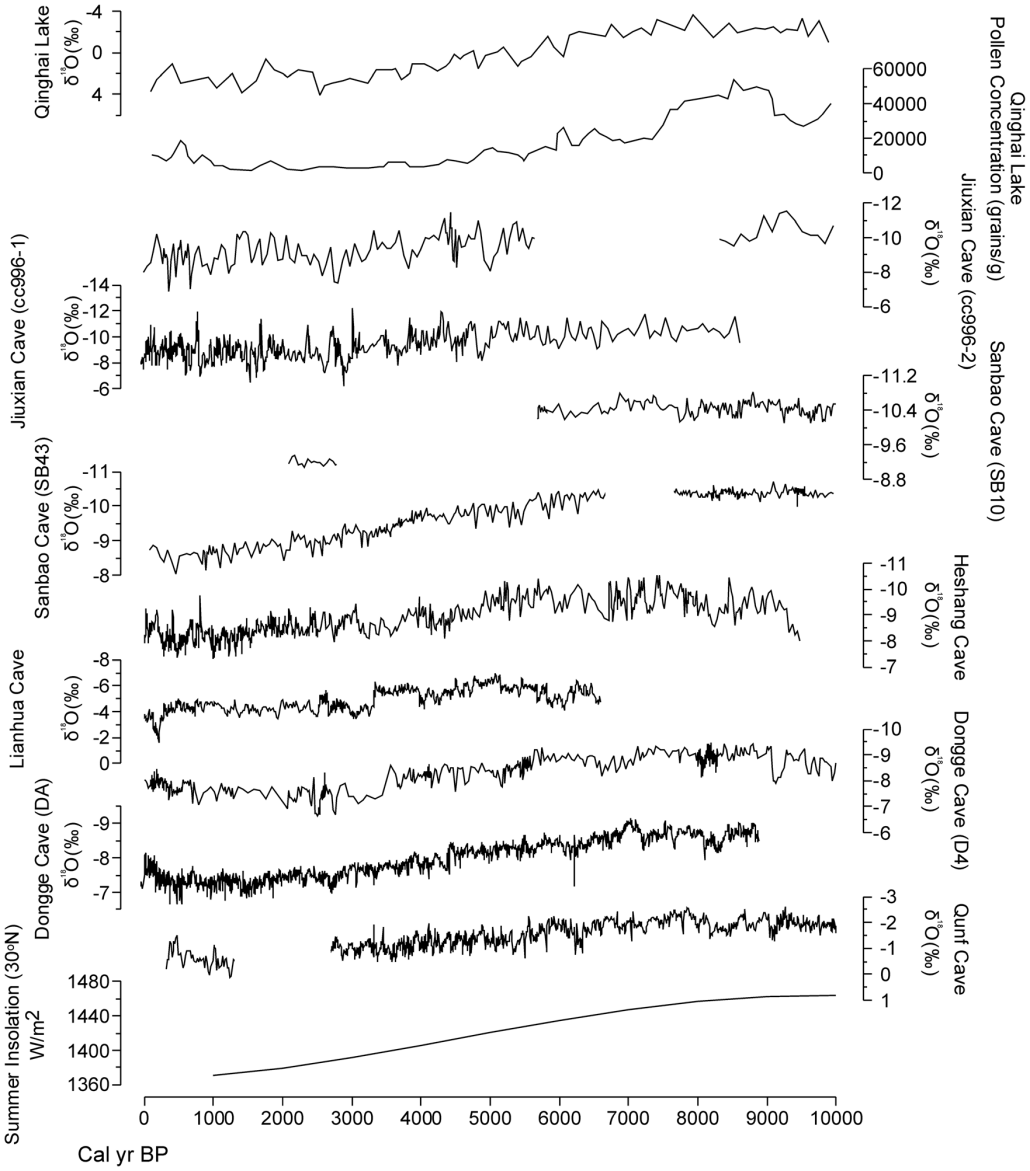
The absolutely dated Holocene speleothem records and the Qinghai Lake record showing the Holocene Asian summer monsoon evolution. The records (from bottom to top) are arranged by latitudes from low to high, which are Summer Insolation at 30°N [50], δ^18^O(‰) for Qunf Cave (17°10′ N)[29], δ^18^O(‰) of DA for Dongge Cave (25°20′ N)[28], δ^18^O(‰) of D4 for Dongge Cave (25°20′ N)[45], δ^18^O(‰) for Lianhua Cave (29°29′ N)[46], δ^18^O(‰) for Heshang Cave (30°27′ N)[47], δ^18^O(‰) of SB43 for Sanbao Cave (31°40′ N)[48], δ^18^O(‰) of SB10 for Sanbao Cave (31°40′ N)[48], δ^18^O(‰) of cc996-1 for Jiuxian Cave (33°34′ N), δ^18^O(‰) of cc996-2 for Jiuxian Cave (33°34′ N)[49], pollen concentration (grains/g) for Qinghai Lake (36°50′ N)[43], δ^18^O(‰) of ostracode shells for Qinghai Lake (36°50′ N)[44].

In the east and south Asian continent, the high-resolution and precisely dated speleothem records provide a reliable Holocene Asian monsoon evolution history, which are widely accepted as an appropriate proxy to reconstruct the Holocene climate change [28], [29]. Although Clemens et al. (2010) argued the cave δ^18^O (‰) cannot be interpreted as reflecting the Asian summer monsoon alone on the orbital scale [41]. During the Holocene epoch, a comparison between speleothem and lake records in the Qinghai-Tibet Plateau and East Asia shows relatively consistent results regarding the Asian summer monsoon evolution [28], [29], [35], [42]–[44]. In this study, we chose speleothem records of Qunf Cave (17°10′ N) [29], Dongge Cave (25°20′ N) [28], [45], Lianhua Cave (29°29′ N) [46], Heshang Cave (30°27′ N) [47], Sanbao Cave (31°40′ N) [48] and Jiuxian Cave (33°34′ N) [49] from low to mid latitudes in East Asia. In addition, Qinghai Lake is the largest saline lake in China and located in the northern Qinghai-Tibet Plateau, where the location is sensitive to advances and retreats of the Asian summer monsoon and a good place for the Holocene Asian summer monsoon reconstruction [43], [44]. The Qinghai Lake (36°50′ N) record is also involved in this study. As shown by the δ^18^O (‰) records in Figure 8, the Asian summer monsoon histories are relatively consistent from south to north and the monsoon intensity reaches the highest level during the early-Holocene (ca. 9000 years ago), and then decreases gradually over the next several thousand years. This pattern closely follows the changes of summer insolation at low latitudes (Figure 8) [50]. In Qinghai Lake, the pollen concentration and δ^18^O (‰) of ostracode shells also show a similar trend, compared with the speleothem records. According to a synthesis of the Asian summer monsoon, the mid-Holocene summer monsoon evolution is relatively stable and showing a trend of gradually decreasing.

### 2 Mid-Holocene lake evolution in arid Central Asia

**Figure 9:**
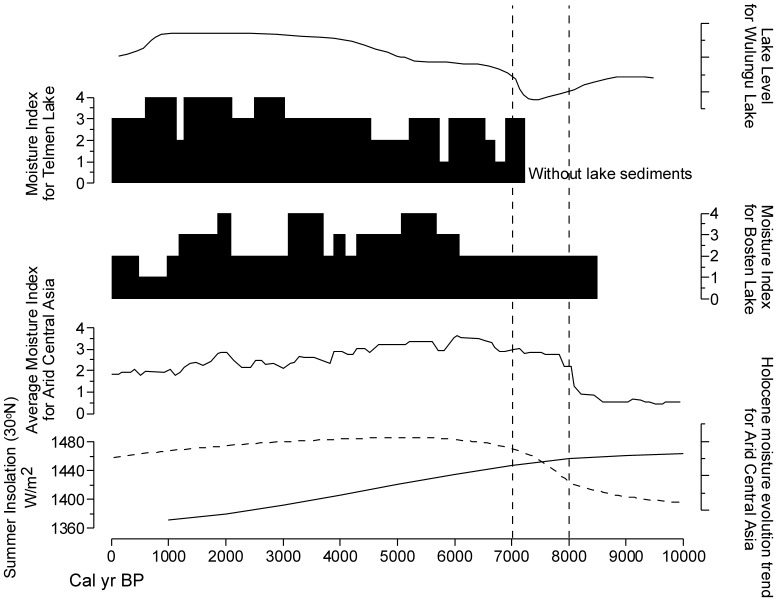
Holocene lake records and moisture indices in arid Central Asia. The two broken lines show the interval between 8.0–7.0 cal kyr BP. From bottom to top, Summer Insolation at 30°N [50], the Holocene moisture evolution trend for arid Central Asia [14], the average moisture index for arid Central Asia [14], the moisture index for Bosten Lake [14], [51], the moisture index for Telmen Lake [14], [52] and the lake level change for Wulungu Lake [53].

In arid Central Asia, Chen et al. (2008) synthesized 11 lake records to evaluate spatial and temporal patterns of moisture changes during the Holocene [14]. The results show that the Holocene moisture change is relatively synchronous and showing a coherent trend: the early-Holocene is relatively dry and the climate becomes wet during the mid-Holocene. In this study, we chose a Holocene moisture evolution trend and a Holocene moisture index of arid Central Asia synthesized by Chen et al. (2008) [14]. Other three lakes, Bosten Lake [14], [51], Telmen Lake [14], [52] and Wulungu Lake [53] were selected for a comparative study. Figure 9 shows a relatively dry early-Holocene and a wet mid-Holocene in arid Central Asia. Between 8.0 and 7.0 cal kyr BP, it is a transitional period between dry and humid phases. As shown in Figure 9, the Holocene summer insolation at 30°N crosses with the Holocene moisture evolution trend of arid Central Asia at 8.0–7.0 cal kyr BP. This period (8.0–7.0 cal kyr BP) is characterized by the gradually decreasing Asian summer monsoon and the relatively arid climate in arid Central Asia. In monsoon marginal zones, the climate is both affected by the Asian summer monsoon and the westerly winds. Therefore, the marginal regions are easily affected by the decreasing monsoon and the arid climate in arid Central Asia, both of which triggered the unstable environment conditions in these regions at 8.0–7.0 cal kyr BP.

### 3 Dry mid-Holocene intervals in monsoon marginal zones

As has been introduced in the Introduction part, there are many dry mid-Holocene records found in the marginal regions of the Asian monsoon. Most of them are recovered from lake records. However, the time scales of the dry events are inconsistent while the explanations regarding the dry mid-Holocene intervals are inconsistent. Generally speaking, the dry mid-Holocene lake records are embedded between early and middle Holocene humid lake records. The early-to-mid Holocene humid environment was interrupted by an abrupt dry interval, which can be seen as a mid-Holocene climate change pattern; although the time scales of the dry mid-Holocene intervals are different. The dating uncertainty can play a very important role for the different time scales. However, in addition to dating errors, how to explain lake records in arid and semi-arid regions is an important to the issue of different time scales. Lake evolution and proxies in arid areas are actually affected by the effective moisture change in the entire basin, which is controlled by the precipitation, evapotranspiration, lake surface evaporation, etc. Li and Morrill (2010) have confirmed the effects of evapotranspiration and lake surface evaporation to lake level changes in East Asia [9]. Various drainage basins are located in monsoon marginal zones and the geographical settings (e.g. topography, geomorphology, soil, vegetation, and hydrology) for different basins vary according to their locations. During the same period, the effective moisture changes could differ from different drainage basins due to the different geographical settings. Therefore, it is normal to find the mid-Holocene dry intervals are asynchronous. In this study, the correlation between the abrupt dry interval in the Shiyang River drainage basin and the long term evolutions of the Asian monsoon and westerly winds provide a clue for detecting the mechanism of the dry mid-Holocene events. The mid-Holocene climate change pattern that the early-to-mid Holocene humid period was interrupted by a centennial-scale abrupt dry interval between 8.0–7.0 cal kyr BP could influence other parts of monsoon marginal zones; however, this mid-Holocene climate pattern could have different time scales due to various effective moisture change patterns, which are related to the geographical settings of East Asia.

## Conclusions

Mid-Holocene climate records were recovered from the terminal lake and middle reaches of the Shiyang River drainage basin. A centennial-scale sand layer was found from QTH01, QTH02 and JDT sections. According to grain size data, the sand layer embedded between lacustrine sediments is related to an abrupt decline in lake level and arid environment in the basin. A comparison between radiocarbon and OSL ages shows the abrupt dry mid-Holocene interval happened between 8.0 and 7.0 cal kyr BP. The mid-Holocene climate record was compared with the long-term Asian monsoon evolution and the moisture history in arid Central Asia. Based on the comparative study, the abrupt centennial-scale dry interval can be related to the long term evolutions of the Asian summer monsoon and the westerly winds. Along with the Holocene insolation change for the low-latitude regions of the Northern Hemisphere, the Asian summer monsoon shows a weakening tendency since the early-Holocene (ca. 9000 years ago). In arid Central Asia, the lake records show a climate transition around 8.0–7.0 cal kyr BP, which indicates a climatic shift from arid to humid. In monsoon marginal zones, the abrupt centennial-scale dry mid-Holocene interval can be influenced by the weakening Asian summer monsoon and relatively arid westerly winds between 8.0–7.0 cal kyr BP. Dry mid-Holocene lake records are widely distributed in monsoon marginal zones. The humid early-to-middle Holocene interrupted by an abrupt dry interval can be seen as a mid-Holocene climate change pattern. The mid-Holocene climate change pattern also can be affected by the long term evolutions of the Asian summer monsoon and the westerly winds. Dating uncertainties can take an important role for different time scales of those dry mid-Holocene intervals. In addition, lake evolution is mainly controlled by the effective moisture change in the basin, while the effective moisture is related to the basin-wide geographical settings. In semi-arid and arid regions, the effective moisture changes vary according to their locations due to various geographical features. The different effective moisture history is another reason for the different time scales of the dry mid-Holocene intervals.
